# PGE2-EP3 signaling pathway contributes to protective effects of misoprostol on cerebral injury in APP/PS1 mice

**DOI:** 10.18632/oncotarget.8284

**Published:** 2016-03-23

**Authors:** Xiaoyan Tian, Chaonan Ji, Ying Luo, Yang Yang, Shengnan Kuang, Shaoshan Mai, Jie Ma, Junqing Yang

**Affiliations:** ^1^ Department of Pharmacology, Chongqing Medical University, The Key Laboratory of Biochemistry and Molecular Pharmacology, Chongqing 400016, China

**Keywords:** Alzheimer's disease, misoprostol, APP/PS1, mPGES-1 - PGE2 - EP signaling pathway, oxidative stress

## Abstract

Epidemiological studies indicate chronic use of non-steroidal anti-inflammatory drugs (NSAIDs), which inhibit the enzymatic activity of the inflammatory cyclooxygenases (COX), reduces the risk of developing Alzheimer's disease (AD) in normal aging populations. Considering multiple adverse side effects of NSAIDs, findings suggest that COX downstream prostaglandin signaling function in the pre-clinical development of AD. Our previous study found that misoprostol, a synthetic prostaglandin E2 (PGE2) receptor agonist, has neuroprotection against brain injury induced by chronic aluminum overload. Here, we investigated the neuroprotective effects and mechanisms of misoprostol on neurodegeneration in overexpressing both amyloid precursor protein (APP) and mutant presenilin 1 (PS1) mice. Here were young group, elderly group, APP/PS1 group and misoprostol-treated group. Mice in misoprostol-treated group were administrated with misoprostol (200 μg·kg^−1^·d^−1^, p.o.) five days a week for 20 weeks. The spatial learning and memory function was impaired and karyopycnosis of hippocampal and cortical neurons was observed; amyloid beta (Aβ) deposition was increased; superoxide dismutase (SOD) activity was decreased and malondialdehyde (MDA) content was increased in APP/PS1 mice. However, misoprostol could significantly blunte these changes in APP/PS1 mic. Moreover, the expressions of microsomal PGE2 synthase (mPGES-1), PGE2, PGE2 receptor (EP) 2 and EP4 were increased and EP3 expression was decreased in APP/PS1 mice, while misoprostol reversed these changes. Our present experimental results indicate that misoprostol has a neuroprotective effect on brain injury and neurodegeneration of APP/PS1 mice and that the activation of PGE2-EP3 signaling and inhibition of oxidative stress contribute to the neuroprotective mechanisms of misoprostol.

## INTRODUCTION

Alzheimer's disease (AD) is a chronic, progressive, age-dependent neurodegenerative disorder and the most common cause of dementia in the elderly [1]. As populations age, AD presents an enormous threat to public health and quality of life [2]. The two major neuropathological hallmarks of AD are extracellular amyloid beta (Aβ) plaques and intracellular neurofibrillary tangles (NFTs) [3]. Currently, there is no specific preventive method and the neurodegenerative mechanism at the basis of AD pathogenesis is still unclear. Many studies showed that inflammation and oxidative stress correlate to AD and other related neurological diseases [4, 5]. Epidemiological studies also indicate that chronic use of non-steroidal anti-inflammatory drugs (NSAIDs) reduces the risk of developing AD in normal aging populations [6, 7]. A primary action of NSAIDs is enzymatic inhibition of cyclooxygenases (COX)-1 and COX-2 activity which further leads to an inhibition of downstream prostaglandin signaling and also exerts multiple adverse side effects mainly characterized by gastrointestinal bleeding and cardiovascular events [8, 9]. In prostaglandin (PG) signaling pathway, tissue-specific terminal prostaglandin synthases or isomerases convert PGH2 into biologically active prostaglandins (PGs), namely, PGD2, PGE2, PGF2a, and PGI2 (also known as prostacyclin), as well as thromboxane A2 (TxA2) [10]. PGE2 is the most versatile prostanoid because of its receptors, PGE2 receptor (EP) subtypes 1 through 4 with characteristic of biological heterogeneity and differential expression in neurons and glial cells throughout the central nervous system [11, 12]. Thus, further studies are needed to target exploitation of downstream prostaglandin signaling pathways. Specifically, targeting individual prostaglandin receptors, rather than inhibiting the entire PG pathway through the use of NSAIDs, offers significant therapeutic benefits for AD while minimizing adverse side effects.

Misoprostol, a synthetic PGE2 analog and PGE2 receptor agonist, has a broad array of therapeutic applications such as prevention and treatment of gastric ulcers induction of uterine contractions, and medical termination of pregnancy [13]. Recently, Li et al. found that administration of misoprostol significantly reduced stroke volume and improved neurological scores in the murine middle cerebral artery occlusion-reperfusion (MCAO-RP) model [14]. Our previous study also found that misoprostol (120 μg.kg^−1^) has a significant protective effect on brain injury and neurodegeneration induced by chronic aluminum overload in rats [15]. Mutant amyloid precursor protein and presenilin 1 (APP/PS1) mouse is a transgenic mouse models for Familial Alzheimer's disease (FAD) overexpressing both APP and PS1. These transgenic mice develop age-dependent accumulation of Aβ along with significant microglial activation [16, 17]. In present study, we used APP/PS1 transgenic mice as animal model to investigate the function of PGE2-EP3 signaling and the neuroprotective effect of misoprostol and to explore the potential mechanisms of AD.

## RESULTS

### Genotype identification of APP/PS1 transgenic mice

**Figure 1:**
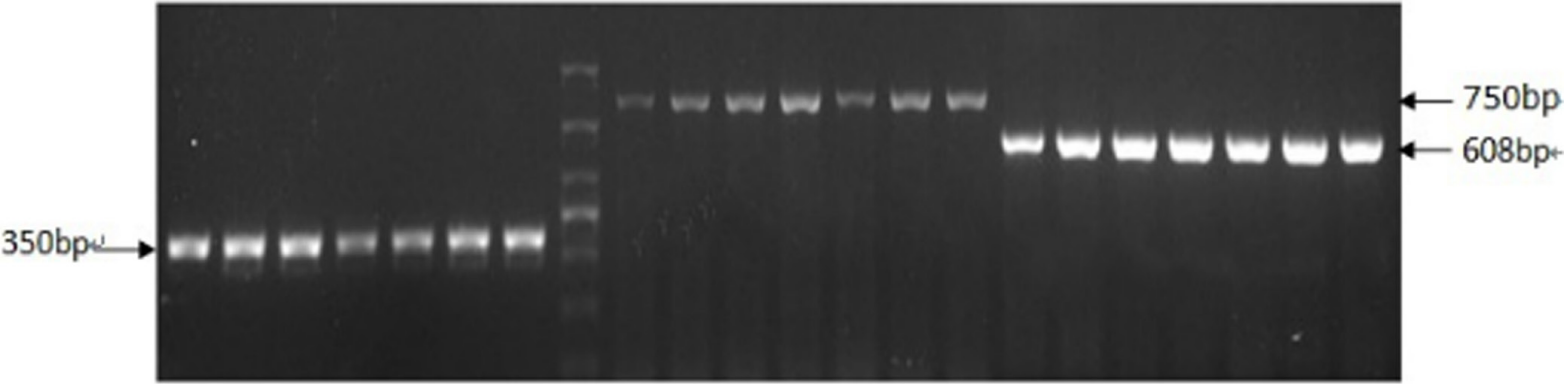
Positive results of APP/PS1 transgenic mice genotype by RT-PCR The RT-PCR product bands of APP gene (350 bp) and PS1 gene (608 bp) were identified clearly.

The RT-PCR product bands of APP gene (350 bp) and PS1 gene (608 bp) were identified clearly (Figure 1). According to the result of RT-PCR, the genotypes of the resulting F2 generation mice were identified as APP/PS1 transgenic mice.

### Changes of spatial learning and memory (SLM) function

**Figure 2:**
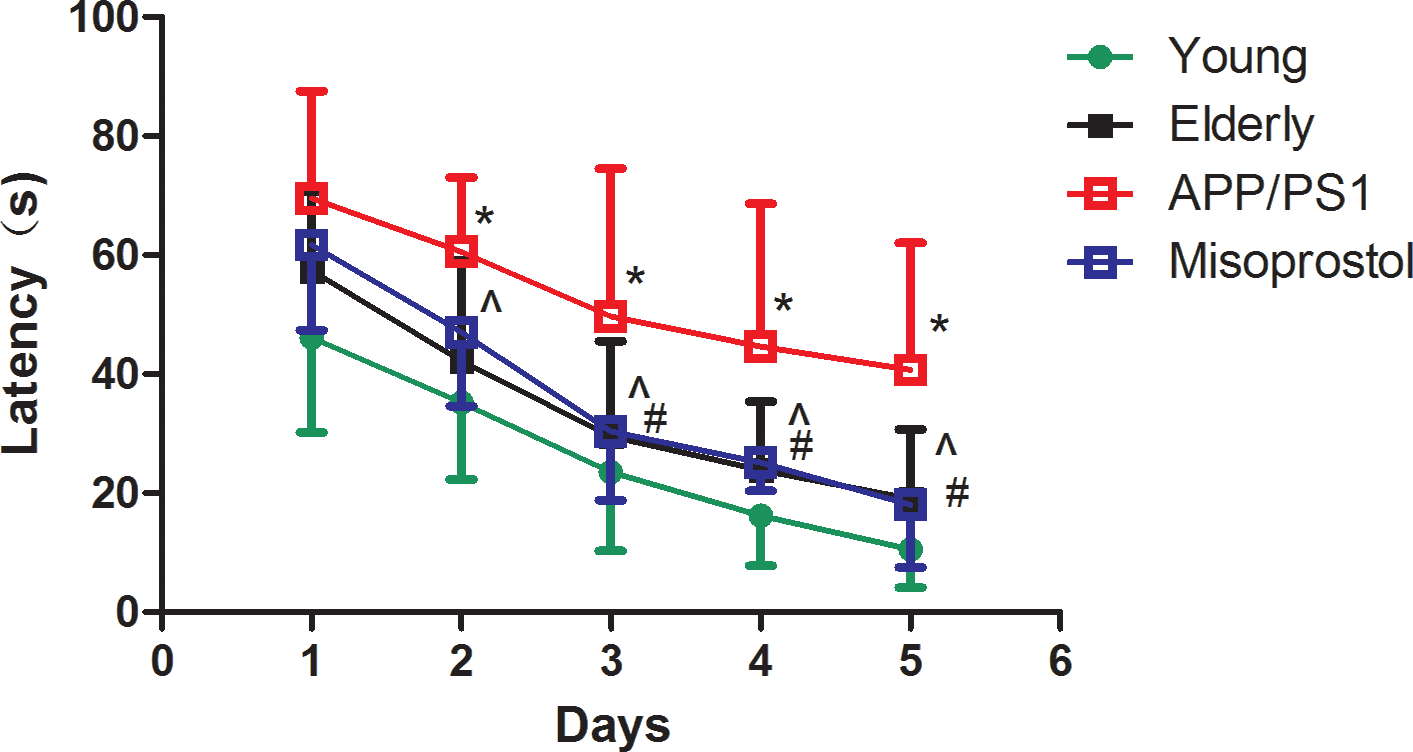
Changes of spatial learning and memory (SLM) function in APP/PS1 mice Data are expressed as mean ± SD of ten individual experiments. ^#^*P* < 0.05, compared with young group, *P < 0.05, compared with elderly group; ^^^*P* < 0.05, compared with APP/PS1 group, respectively.

Mice were trained for 4 days to learn the location of a hidden platform, and the time required to reach the platform (latency) was measured. The latency from d3 to d4 in elderly group was significantly longer compared with young group (^#^*P* < 0.05). The latency from d2 to d4 in APP/PS1 group was significantly longer compared with elderly group (*P < 0.05). The latency from d2 to d4 in misoprostol-treated group was significantly shortened compared with APP/PS1 group (^^^*P* < 0.05). For the memory function of mice, the latency in elderly group was significantly longer compared with young group (^#^*P* < 0.05). The latency in APP/PS1 group was significantly longer compared with elderly group (*P < 0.05). The latency in misoprostol-treated group was significantly shortened compared with APP/PS1 group (^^^*P* < 0.05) (Figure 2).

### Changes in neuronal pathomorphology

**Figure 3:**
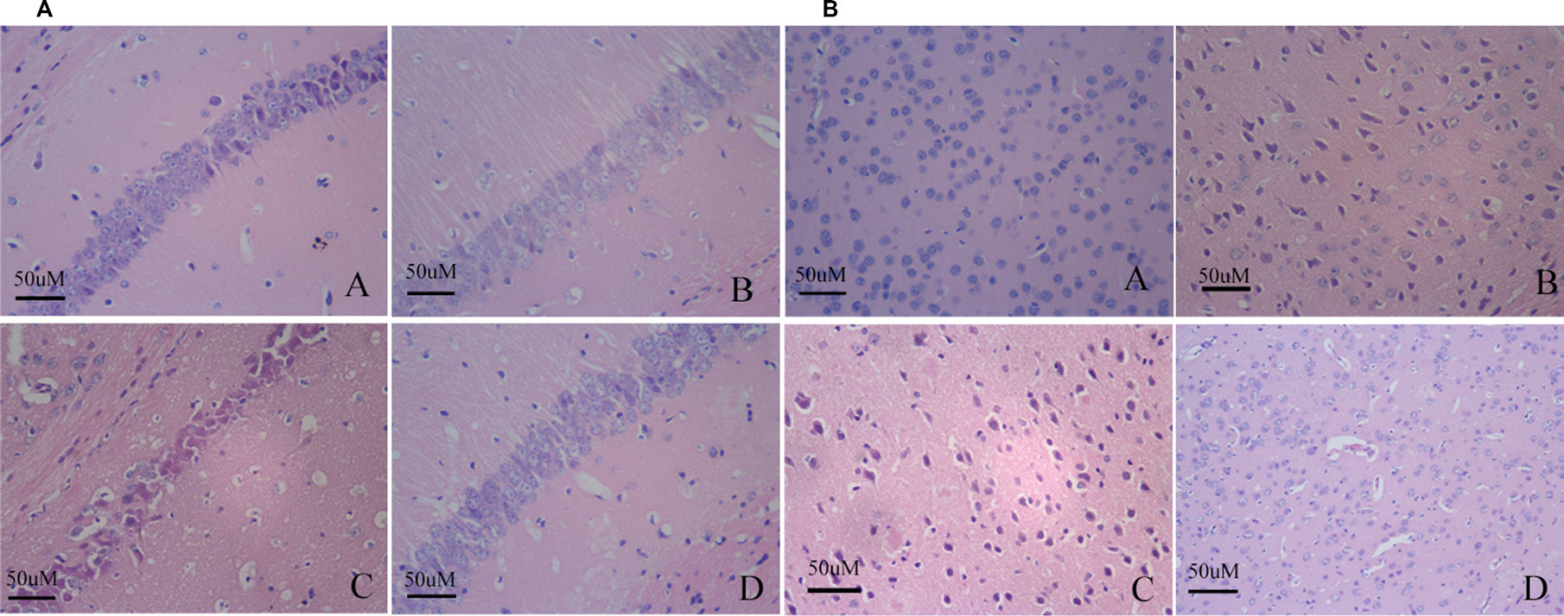
Changes of neuronal pathomorphology in hippocampus and cortex (HE × 400, Scale bars = 50 μm) (**A** and **B**) Changes of neuronal pathomorphology in hippocampus and cortex (HE × 400, Scale bars = 50 μm), respectively. A: Young group; B: Elderly group; C: APP/PS1 group; D: Misoprostol-trearted group. The hippocampal and cortical neurons were in distinct and regular structure, and arranged densely and clearly in young group (Figure 3A). Elderly group revealed injuries including cell loss and karyopycnosis, and hyperchromatic nuclei in hippocampal and cortical neurons (Figure 3B). Compared to elderly group, APP/PS1 group revealed significant injuries including remarkable cell loss and karyopycnosis, and hyperchromatic nuclei in hippocampal and cortical neurons. Dead and dying cells in the injured hippocampi and cortex displayed necrosis, karyopycnosis and irregular contours (Figure 3C). Misoprostol-treated group significantly reduced injuries including cell loss and karyopycnosis, and hyperchromatic nuclei in hippocampal and cortical neurons (Figure 3D).

The hippocampal and cortical neurons were in distinct and regular structure, and arranged densely and clearly in young group. In contrast, elderly group revealed injuries including cell loss and karyopycnosis, and hyperchromatic nuclei in hippocampal and cortical neurons, suggesting that elderly group showed serious pathological changes with age-dependent manner. Compared to elderly group, APP/PS1 group revealed significant injuries including remarkable cell loss and karyopycnosis, and hyperchromatic nuclei in hippocampal and cortical neurons. Dead and dying cells in the injured hippocampi and cortex displayed necrosis, karyopycnosis and irregular contours. Misoprostol-treated group significantly reduced injuries including cell loss and karyopycnosis, and hyperchromatic nuclei in hippocampal and cortical neurons (Figure 3).

### Changes in Aβ deposition

**Figure 4:**
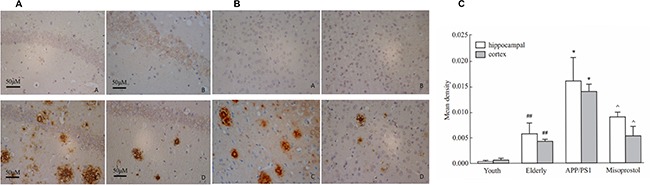
Changes of Aβ deposition in hippocampus and cortex (× 400, Scale bars = 50 μm) (**A** and **B**): Changes of Aβ deposition in hippocampus and cortex (× 400, Scale bars = 50 μm), respectively. A: Young group; B: Elderly group; C: APP/PS1 group; D: Misoprostol-trearted group. Both hippocampal and cortex Aβ deposition in elderly group distinctly increased compared with young group. Both hippocampal and cortex Aβ deposition in APP/PS1 group distinctly increased compared with elderly group. Misoprostol significantly reversed the increase of hippocampal and cortex Aβ deposition. (**C**) Group data showing the change of Aβ deposition. Data are expressed as mean ± SD of four individual experiments. ^#^*P* < 0.05, compared with young group; *P < 0.05, compared with elderly group; ^^^*P* < 0.05, compared with APP/PS1 group.

Immunohistochemistry was performed on brain sections to evaluate Aβ deposition. Young group exhibited less Aβ deposition both in the cortex and hippocampus neurons compared with elderly group. In APP/PS1 group, the Aβ positive plaques were distributed in intracellular and extracellular spaces in spherical or irregular shapes with different sizes. The Aβ deposition in the cerebral cortex and hippocampus in misoprostol-treated group was significantly lower than that in APP/PS1 group (Figure 4).

### Changes in superoxide dismutase (SOD) activity and malondialdehyde (MDA) content

**Figure 5:**
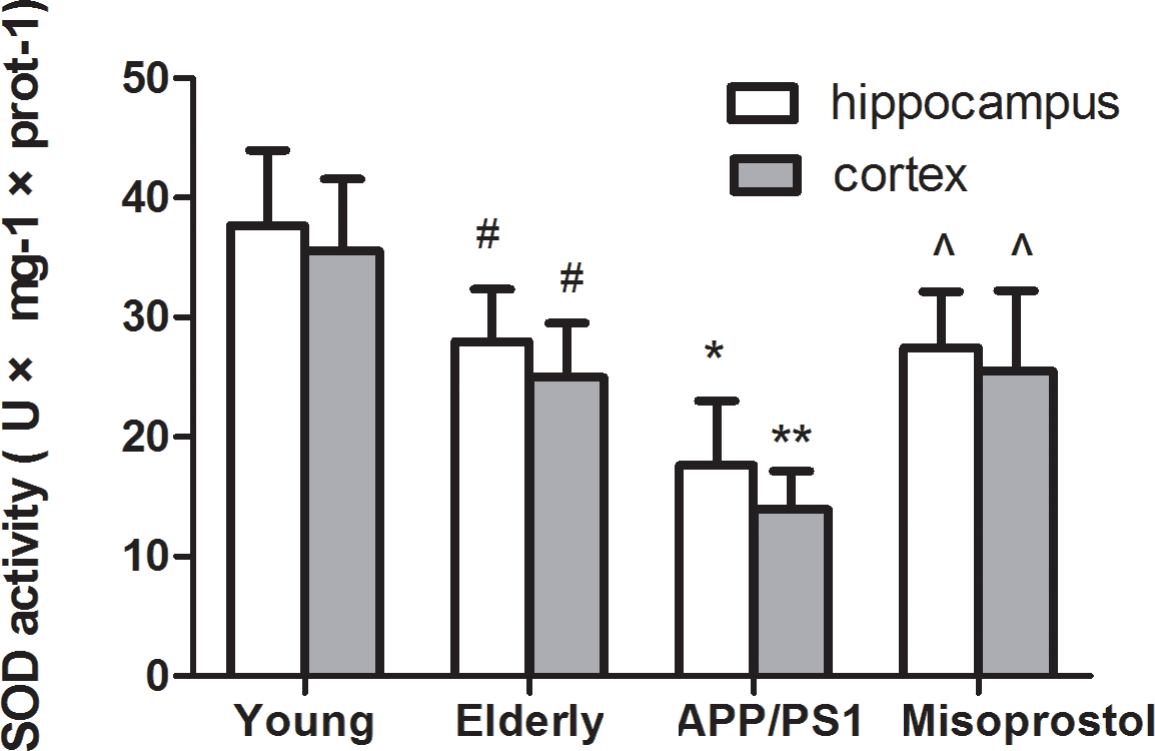
Changes of SOD activities in hippocampus and cortex Data are expressed as mean ± SD of six individual experiments. ^#^*P* < 0.05 and ^##^*P* < 0.01 compared with young group, respectively. *P < 0.05, compared with elderly group. ^^^*P* < 0.05, compared with APP/PS1 group.

To confirm the effect of oxidative stress in AD, we tested SOD activity and MDA content in hippocampi and cortex. Both hippocampal and cortex SOD activity in elderly group distinctly decreased compared with young group. Both hippocampal and cortex SOD activity in APP/PS1 group distinctly decreased compared with elderly group. Misoprostol administration significantly reversed the decrease of SOD activity (Figure 5).

**Figure 6:**
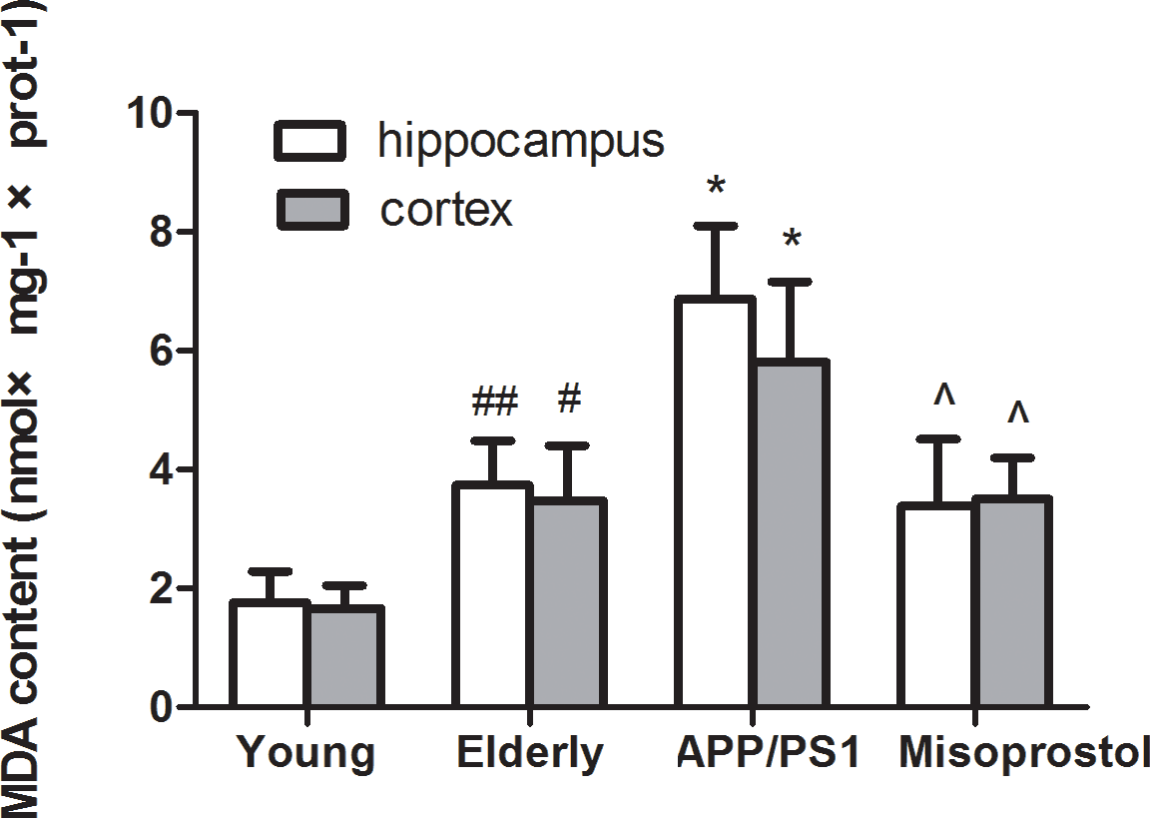
Changes of MDA contents in hippocampus and cortex Data are expressed as mean ± SD of six individual experiments. ^#^*P* < 0.05, compared with young group. *P < 0.05 and **P < 0.01 compared with elderly group, respectively. ^^^*P* < 0.05, compared with APP/PS1 group.

Both hippocampal and cortex MDA content in elderly group distinctly increased compared with young group. Both hippocampal and cortex MDA content in APP/PS1 group significantly increased compared to elderly group. Misoprostol administration significantly blunted the increase of MDA content in APP/PS1 mice (Figure 6).

### Changes in microsomal PGE2 synthase (mPGES-1) expression, PGE2 content and EPs expressions

To confirm the effect of downstream prostaglandin signaling in AD, we tested changes in mPGES-1 expression, PGE2 content and EP_1–4_ expressions.

**Figure 7:**
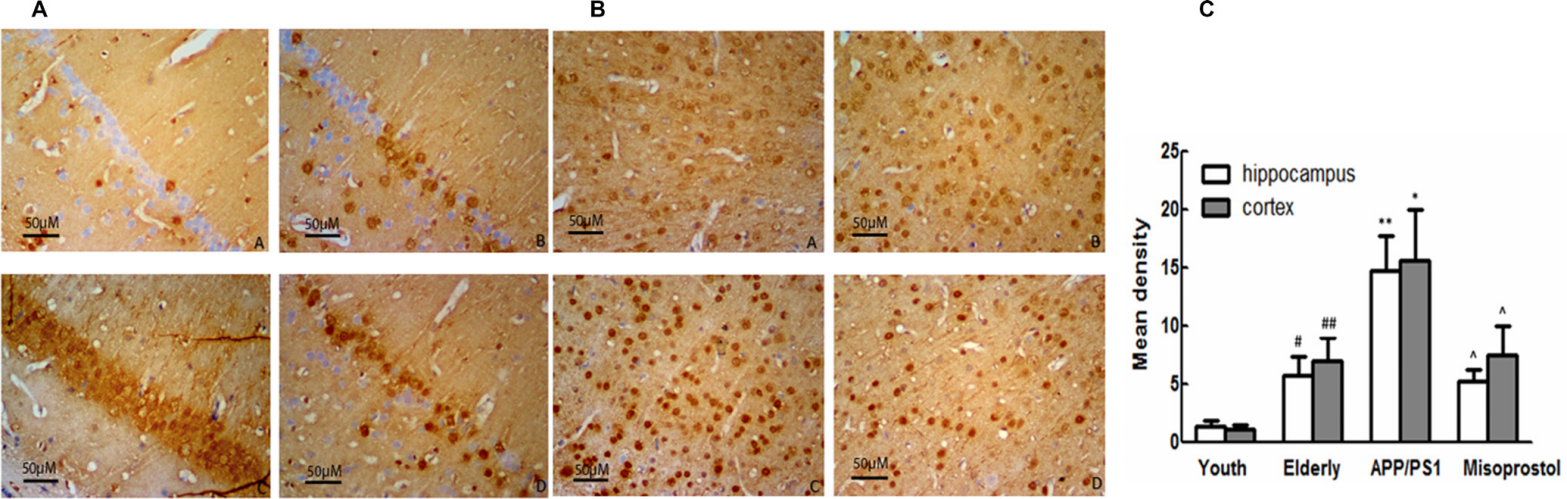
Changes of mPGES-1 expression in hippocampus and cortex (× 400) (**A** and **B**): Changes of mPGES-1 expression in hippocampus and cortex (× 400, Scale bars = 50 μm), respectively. A: Young group; B: Elderly group; (**C**) APP/PS1 group; D: Misoprostol-trearted group. Both hippocampal and cortex mPGES-1 expression in elderly group distinctly increased compared with young group. Both hippocampal and cortex mPGES-1 expression in APP/PS1 group distinctly increased compared with elderly group. Misoprostol significantly reversed the increase of hippocampal and cortex mPGES-1 expression. C: Group data showing the change of mPGES-1 expression. Data are expressed as mean ± SD of four individual experiments. ^#^*P* < 0.05 and ^##^*P* < 0.01 compared with young group, respectively; *P < 0.05 and **P < 0.01 compared with elderly group, respectively; ^^^*P* < 0.05, compared with APP/PS1 group.

Both hippocampal and cortex mPGES-1 expression of mPGES-1 in elderly group distinctly increased compared with young group. Both hippocampal and cortex mPGES-1 expression in APP/PS1 group distinctly increased compared with elderly group. APP/PS1 mice treated with misoprostol showed a significant decrease of hippocampal and cortex mPGES-1 expression (Figure 7).

**Figure 8:**
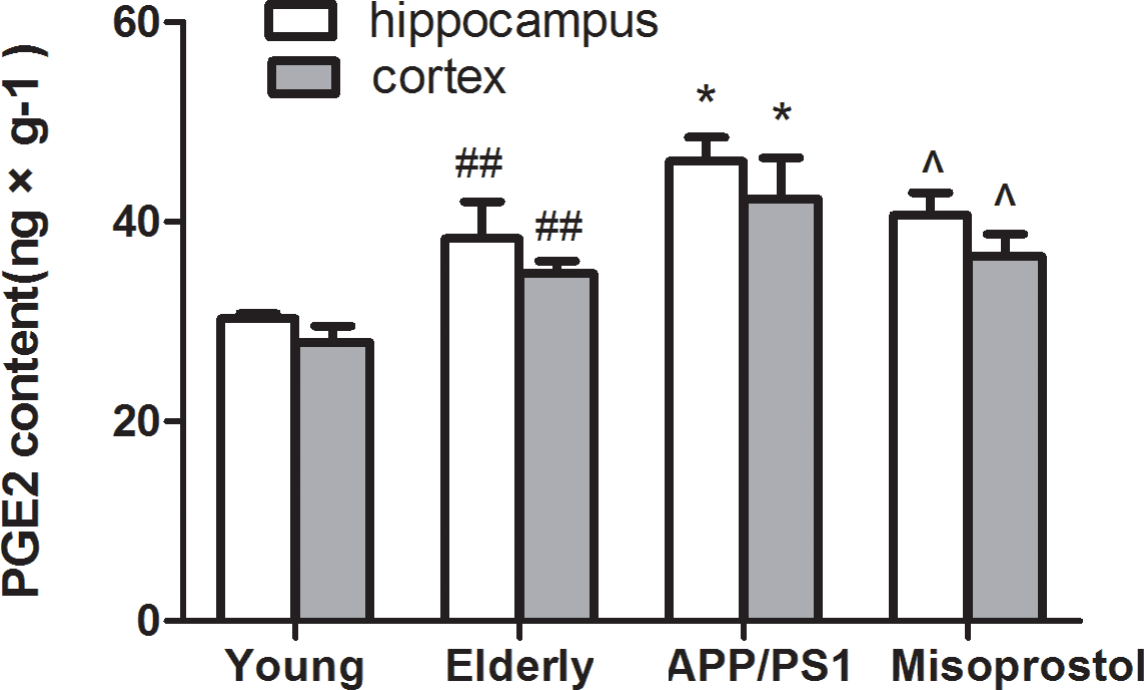
Changes of PGE^2^ contents in hippocampus and cortex Data are expressed as mean ± SD of six individual experiments. ^##^*P* < 0.01, compared with young group. *P < 0.05, compared with elderly group. ^^^*P* < 0.05, compared with APP/PS1 group.

PGE2 content in elderly group distinctly increased compared with young group. PGE2 content in APP/PS1 group significantly increased compared to elderly group. Misoprostol administration significantly blunted the increase of PGE2 content in APP/PS1 mice (Figure 8).

**Figure 9:**
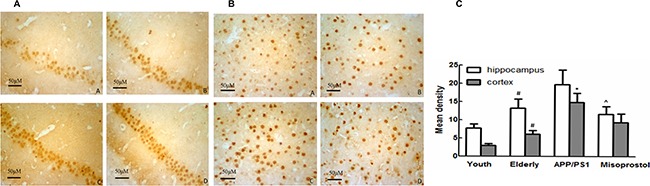
Changes of EP1 expression in hippocampus and cortex (× 400) (**A** and **B**) Changes of EP1 expression in hippocampus and cortex (× 400, Scale bars = 50 μm), respectively. A: Young group; B: Elderly group; C: APP/PS1 group; D: Misoprostol-trearted group. EP_1_ expression of mice brain in elderly group distinctly increased compared with young group. The increase of hippocampal EP_1_ expression in APP/PS1 group was not significant compared with elderly group. EP_1_ expression in misoprostol-treated group significantly decreased. Cortex EP_1_ expression in APP/PS1 group was significantly increased compared with elderly group; EP_1_ expression in misoprostol-treated group did not significantly decrease. (**C**) Group data showing the change of EP1 expression. Data are expressed as mean ± SD of four individual experiments. ^#^*P* < 0.05, compared with young group. *P < 0.05, compared with elderly group. ^^^*P* < 0.05, compared with APP/PS1 group.

EP_1_ expression of mice hippocampi and cortex in elderly group distinctly increased compared with young group. The increase of hippocampal EP_1_ expression in APP/PS1 group was not significant compared with elderly group. Misoprostol treatment significantly decreased EP_1_ expression. Cortex EP_1_ expression in APP/PS1 group was significantly increased compared with elderly group; Misoprostol treatment did not significantly decrease cortex EP_1_ expression (Figure 9).

**Figure 10:**
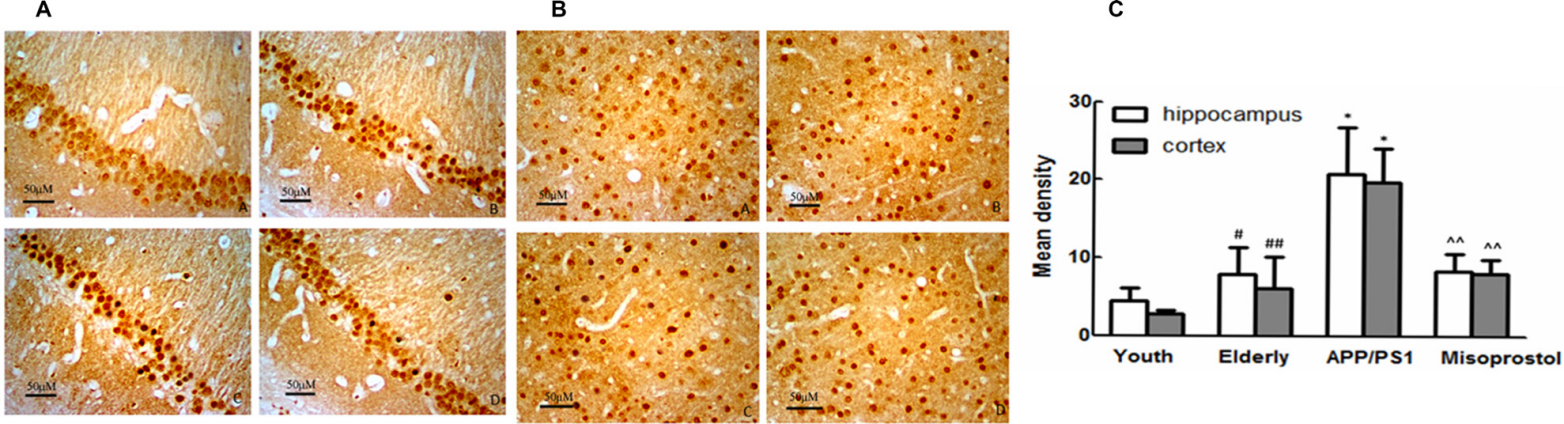
Changes of EP2 expression in hippocampus and cortex (× 400) (**A** and **B**) Changes of EP2 expression in APP/PS1 mice hippocampus and cortex (× 400, Scale bars = 50 μm), respectively. A: Young group; B: Elderly group; C: APP/PS1 group; D: Misoprostol-trearted group. EP_2_ expressions of mice brain in elderly group distinctly increased compared with young group. EP_2_ expressions in APP/PS1 group distinctly increased compared with elderly group. Misoprostol significantly reversed the increase of EP_2_. (**C**) Group data showing the change of EP2 expression. Data are expressed as mean ± SD of four individual experiments. ^#^*P* < 0.05 and ^##^*P* < 0.01 compared with young group, respectively; *P < 0.05, compared with elderly group; ^^^^*P* < 0.01 compared with APP/PS1 group.

EP_2_ and EP_4_ expressions of mice hippocampi and cortex in elderly group distinctly increased compared with young group. EP_2_ and EP_4_ expressions in APP/PS1 group distinctly increased compared with elderly group. APP/PS1 mice treated with misoprostol showed the significant decrease of EP_2_ and EP_4_ expressions (Figure 10 and Figure 12).

**Figure 11:**
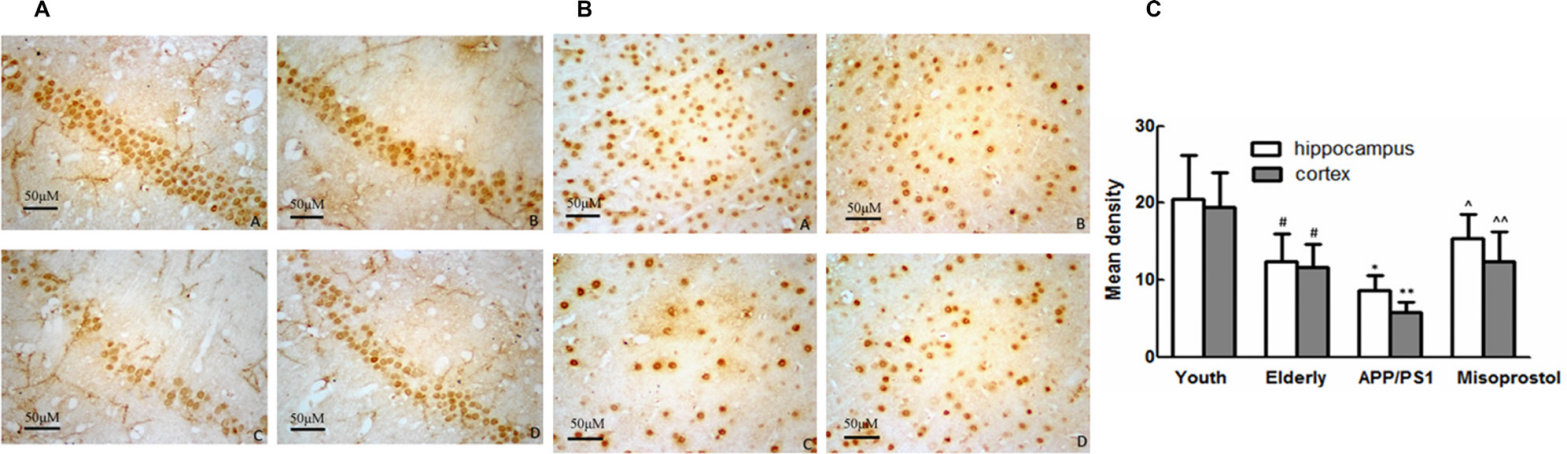
Changes of EP3 expression in hippocampus and cortex (× 400) (**A** and **B**) Changes of EP3 expression in hippocampus and cortex (× 400, Scale bars = 50 μm), respectively. A: Young group; B: Elderly group; C: APP/PS1 group; D: Misoprostol-trearted group. EP_3_ expression of mice brain in elderly group distinctly decreased compared with young group. EP_3_ expression in APP/PS1 group distinctly decreased compared with elderly group. Misoprostol significantly reversed the decrease of EP_3_ expression. (**C**) Group data showing the change of EP3 expression. Data are expressed as mean ± SD of four individual experiments. ^#^*P* < 0.05, compared with young group; *P < 0.05 and **P < 0.01 compared with elderly group, respectively; ^^^*P* < 0.05 and ^^^^*P* < 0.01 compared with APP/PS1 group, respectively.

EP_3_ expression of mice hippocampi and cortex in elderly group distinctly decreased compared with young group. EP_3_ expression in APP/PS1 group distinctly decreased compared with elderly group. APP/PS1 mice treated with misoprostol showed a significant increase of EP_2_ expression (Figure 11).

**Figure 12:**
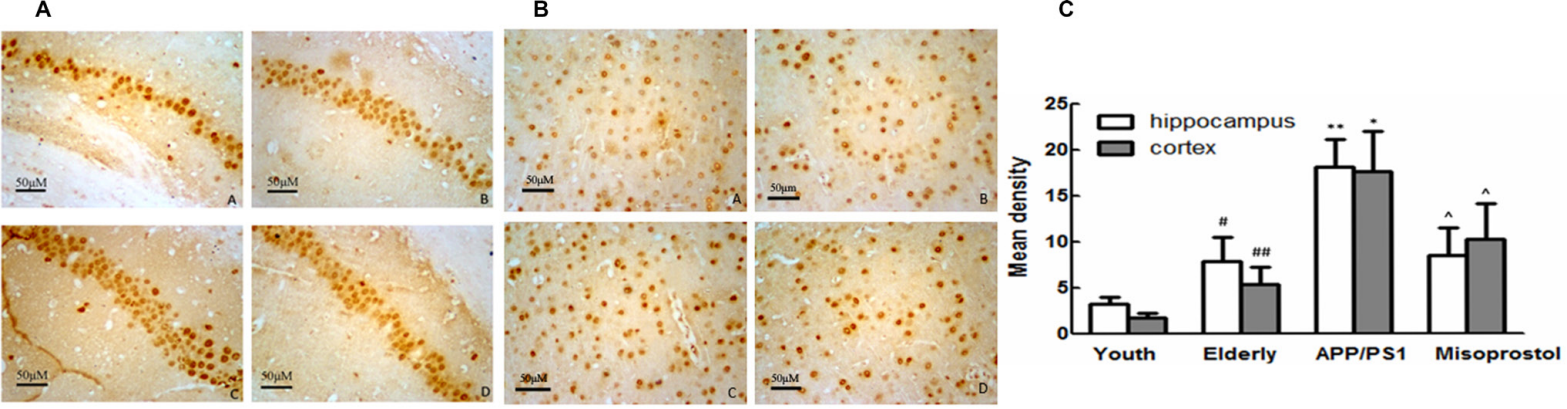
Changes of EP4 expression in hippocampus and cortex (× 400) (**A** and **B**) Changes of EP4 expression in hippocampus and cortex (× 400, Scale bars = 50 μm), respectively. A: Young group; B: Elderly group; C:APP/PS1 group; D: Misoprostol-trearted group. EP_4_ expressions of mice brain in elderly group distinctly increased compared with young group. EP_4_ expressions in APP/PS1 group distinctly increased compared with elderly group. Misoprostol significantly reversed the increase of EP_4_. (**C**) Group data showing the change of EP4 expression. Data are expressed as mean ± SD of four individual experiments. ^#^*P* < 0.05 and ^##^*P* < 0.01 compared with young group, respectively; *P < 0.05 and **P < 0.01 compared with elderly group, respectively; ^^^*P* < 0.05, compared with APP/PS1 group.

## DISCUSSION

Alzheimer's disease is the most common cause of dementia in the elderly and has insidious onset, chronic progression and long duration [18]. Although, to date, several independent hypotheses have been proposed to explain the disease, pharmacological treatments of Alzheimer's disease are limited. Thus, further studies are needed to target mechanism of AD and develop specific preventive drug.

In the present study, SLM function was significantly decreased, karyopycnosis and hyperchromatic nuclei in hippocampal and cortical neurons were serious, Aβ deposition was increased and formed senile plaques in APP/PS1 mice, which is consistent with the results of Wang et al. [19].

Increasing evidence suggests that oxidative stress is a prominent and early feature of AD and correlate to its pathogenesis. SOD are the most important antioxidant enzymes in the antioxidant defense system. MDA is an end-product of lipid peroxidation and an excellent marker for degeneration of neurons [20, 21]. Our experimental results showed that, in APP/PS1 group, SOD activity distinctly decreased and MDA content significantly increased compared with elderly group. Treatment with misoprostol significantly reversed the decrease of SOD activity and the increase of MDA content, decreased Aβ deposition and the number of senile plaques, reduced cerebral karyopycnosis and hyperchromatic nuclei and shortened the latency of APP/PS1 mice. The results suggested that misoprostol administration could reduce oxidative stress of brain and injury of neurons in APP/PS1 mice and improved SLM function. Similarly, Abdel et al. found that misoprostol can alleviate oxidative stress in the brain and exert neuroprotective effect through decreasing cerebral MDA content and increasing cerebral GSH activity during systemic inflammation induced by lipopolysaccharide (LPS) [22]. Our results might further confirm the hypothesis that neuronal protection of misoprostol in APP/PS1 mice is involved in occurrence of oxidative stress.

In prostaglandin signaling pathway, PGE2 is the major effector in the CNS based on synthase expression data and direct measurements, and is the most studied with regard to neuroinflammation [23]. PGE2 is synthesized in either the membrane by microsomal prostaglandin E synthase (mPGES) or in the cytosol by cytosolic PGES (cPGES). As the inducible form, much attention has been paid to the value of mPGES as a potential therapeutic target, and the efficacy of a variety of agonist has been investigated in relevant disease models [24]. Prostaglandin E2 (PGE2) has been described to exert beneficial and detrimental effects in various neurologic disorders. These conflicting roles of PGE2 could be attributed to its diverse receptor subtypes, EP1-EP4 [25]. In our study, mPGES-1 and PGE_2_ expressions in APP/PS1 group significantly increased compared with elderly group. Treatment with misoprostol significantly reversed the increase of mPGES-1 and PGE_2_ expressions in APP/PS1 mice. The results indicated that mPGES-1 and PGE_2_ are considered to exert neurotoxicity effects in AD, the decrease of mPGES-1 expression results in the decrease of PGE2 expression and may exert partly neuroprotective effect. However, it is important to note that the precision gained through targeted mPGES inhibition is only one level more selective than COX inhibitors. Instead, we propose that greater therapeutic value can be achieved by targeting prostaglandin receptors, the next level down.

It has been shown previously that PGE2 acts on all four EP receptors with varying affinities and predominantly on EP3 and EP4 receptors (EP3 > EP4» EP2 > EP1). According to previous studies misoprostol also acts on EP receptors but with much lower affinities than PGE2 and mainly on the EP3 receptor [26]. We hypothesized that misoprostol, which can bind all three EP receptors and mainly acts on EP3 receptor, might also mediate pharmacological protection on transgenic model of AD.

Studies showed that PGE2 EP1 receptor mainly cause severe neurological and functional deficits in the nervous system diseases [25, 27, 28]. We found that the increase of hippocampal EP_1_ expression in APP/PS1 group was not significant compared with elderly group and misoprostol treatment significantly decreased EP_1_ expression. Cortex EP_1_ expression in APP/PS1 group was significantly increased compared with elderly group; misoprostol had no significant effect on EP_1_ expression. Our results indicated that misoprostol protection was not correlate to the PGE_2_-EP1 pathway and the effect of EP_1_ need to be further investigated.

In our study, EP2 and EP4 expressions in APP/PS1 group significantly increased and EP3 expression in APP/PS1 group significantly decreased compared with elderly group. Treatment with misoprostol significantly reversed the increase of EP2 and EP4 expressions and the decrease of EP3 expression in APP/PS1 mice. These results suggest neurotoxicity roles of EP2 and EP4 in AD. Similarly, Liang et al. found that deletion of the PGE2 EP2 receptor in the APPSwe-PS1E9 model results in marked reductions in lipid peroxidation in aging mice. This reduction in oxidative stress is associated with significant decreases in levels of amyloid- (A) 40 and 42 peptides and amyloid deposition [29]. Hoshino et al. also found that mice lacking the EP4 receptor displayed lower levels of Aβ plaque deposition and less neuronal and synaptic loss than control mice. Oral administration of a specific EP4 receptor antagonist, AE3–208 to APP23 mice, improved their cognitive performance, as well as decreasing brain levels of Aβ and suppressing endocytosis and activation of c-secretase [30].

Up to now, EP3 receptor is considered to exert neuroprotective effects [24]. EP3 is the only EP receptor that has three transcriptional splice variants, EP3α, EP3β, and EP3γ. The EP3β receptor is unique because it does not desensitize and thus displays persistent signaling when opposed to its ligand. EP3 receptor couples to Gi protein and mediates the decrease in cAMP concentration through inhibition of adenylate cyclase, while stimulation of EP2 and EP4 receptors leads to the elevation of cAMP level *via* Gs protein [10, 31]. Due to the complexity mentioned above, the activation of different EP receptors could lead to opposite functions. Vio, C. P. et al. found administration of a selective EP3 receptor antagonist increased the area for COX-2-stained cells and COX-2 mRNA accumulation and suggested that COX-2 levels are regulated by a novel negative feedback loop mediated by PGE2 acting on its EP3 receptor in the thick ascending limb [32]. It indicated that misoprostol as EP3 agonist, couples to EP3 receptor and leads to the decrease in mPGES-1 and PGE2 expression, and further leads to the increase of EP3 expression and the decrease of EP2 and EP4 expression through negative feedback regulation. However, further studies are needed to target at EP3 receptor and its transcriptional splice variants.

Take together, misoprostol as EP3 agonist, couples to EP3 receptor and results in the decrease in mPGES-1 and PGE2 expression, and further leads to the increase of EP3 expression and the decrease of EP2 and EP4 expressions through a negative feedback regulation mechanism. Our findings indicate that PGE2 signaling *via* the EP3 receptor alleviates age-dependent oxidative damage and decreases Aβ deposition in the mouse model of AD and suggest a rationale therapeutic strategy for targeting the EP3 receptor in neuroinflammatory diseases such as AD.

## MATERIALS AND METHODS

### Animals

All the experimental procedures were approved by the Animal Laboratory Administrative Center and the Institutional Ethics Committee at Chongqing Medical University and also in accordance with the National Institutes of Health guidelines. The new transgenic AD APP/PS1 mouse model was obtained by breeding APPswe/PSEN1dE9 AD mice [Animal license number: SCXK (su) 2010–0111], which were purchased from the Model Animal Research Center of Nanjing University. The genotypes of the resulting F2 generation mice were determined by standard polymerase chain reaction (PCR) analysis of mouse tail genomic DNA using the Biospin Tissue PCR Kits (Hangzhou Bioer Technology Co., Ltd.) according to the manufacturer's instructions. Specific primers for APP gene contained within the Sangon Biotech (Shanghai) designed (forward Neo′; 5′-GACTGACCACTCGACCAGGTTCTG-3′ and reverse Neo3′; 5′-CTTGTAAGTTGGATTCTCATATCCG-3′). Also, specific primers for PS1 gene contained within the Sangon biotech (Shanghai) designed (forward Neo5′; 5′-AATAGAGAACGGCAGGAGCA-3′ and reverse Neo3′; 5′-GTGGATAACCCCTCCCCCAGCCTAGACC -3′). After PCR reactions, the amplified products were separated in agarose gels and analyzed. C57/BL6 as wild type were purchased from the Experimental Animal Center of Chongqing Medical University [Animal license number: SCXK (yu) 2012–0001]. The animals were housed in controlled environment (Experimental Animal Center, Chongqing Medical University, temperature 22°C, light7:00–19:00; humidity 50–60%), and food and water were freely available. All behavioral tests were conducted during the light phase (8:00–16:00).

### Protocols

There were 4 groups including young group, elderly group, APP/PS1 group and misoprostol-treated group. Twenty 24-weekth-old APP/PS1 transgenic mice were randomized into two groups as APP/PS1 group and misoprostol-treated group. Ten 24-weekth-old wild-type C57 mice were chosen as elderly group. Mice in misoprostol-treated group were administrated with misoprostol (200 μg·kg^−1^·d^−1^, p.o.) five days a week for 20 weeks. In our previous study, we investigated the protective effect of misoprostol (30, 60 and 120 μg·kg^−1^, p.o.) on neurodegeneration induced by chronic aluminum overload in rats and found that misoprostol (120 μg·kg^−1^) had obvious protective effect. So, according to our previous study, the dosage of misoprostol (200 μg·kg^−1^) for APP/PS1 mouse in present study was designed based on the body surface rate of rat to mouse (Bios's formula) [15]. Mice in APP/PS1 group and elderly group were administrated with carboxymethylcellulose sodium (200 μg·kg^−1^·d^−1^, p.o.) five days a week for 20 weeks. In addition, ten 8-weekth-old wild-type C57 mice were chosen as young group.

### Morris water maze

Morris water maze was used to evaluate spatial learning and memory (SLM) function of mice in each group [33]. We evaluated the morris water maze test at the end of gastric perfusion. The apparatus consisted of a circular pool (120 cm diameter*50 cm height) with a black inner wall and of transparent platform (10 cm diameter) submerged 1 cm below the water surface. The water maintained at 24–25°C. In the learning stages, mice received 4 trials on each of 4 days. In each trial, a mouse was placed into the water facing the pool wall, randomly from each of four starting positions. The trial was terminated and the latency was recorded when the mouse found the platform within 90 s. Otherwise, the trial was terminated and the mouse was led to the platform. On the fifth day, the mice received a probe trial in which the platform was removed to evaluate the memory function of mouse. The mouse was placed into the water as before to test its memory about the previous position of the platform. The time for a mouse to pass through the place of the platform was recorded as the latency.

### Histology

After the Morris water maze test, 4 mice from each group were perfused with heparinized saline (30 ml) to remove blood from the vasculature, and then with 4% paraformaldehyde in phosphate buffered saline (50 ml). The whole brain was then removed and stored in the same fixative. After paraffin embedding, 5-μm sections were obtained and stained with hematoxylin-eosin (H & E) [34]. Morphologic changes of hippocampal and cortical neurons were examined using light microscopy. High power fields were sampled from the hippocampal CA1 subfield. Cells with a distinct nucleus and nucleolus were regarded as intact neurons.

### Immunohistochemistry

Immunohistochemistry was performed to investigate the expression of Aβ, mPGES-1 and EP1, 2, 3, 4 in the mice brains. Briefly, brains sections of 4 mice from each group were dewaxed and rehydrated in decreasing concentrations ethanol. Then, endogenous peroxidase of the sections were blocked for with 30 g.L^−1^ H_2_O_2_ under room temperature for 15 minutes. Slides were washed with PBS for several times and pre-incubated in 5% normal goat serum for 30 min at 37°C. Thereafter, the sections were incubated with primary antibodies mPGES-1 (dilution 1:50, Santa, USA), Aβ (1:100, Abcam, Cam-bridge, UK) and EP1, 2, 3, 4 (dilution EP1, 1:100; EP2, 1:200; EP3, 1:100; EP4, 1:50, Santa, USA) overnight at 4°C. Then, the sections were incubated with biotinylated secondary antibody (dilution 1:100) for 30 min at 37°C, and incubated with streptavidin for 20 min, and then rinsed for another 3 min × 3 with PBS before reaction with DAB solution. The sections were counterstained with hematoxylin and then observed under a microscope. High power fields were sampled from the hippocampal CA1 subfield.

### Measurement of SOD activity and MDA content

After Morris water maze test, 6 mice brains from each group were harvested and isolated hippocampi and cortex. The hippocampi and cortex were homogenized with normal saline. Then SOD activity was detected using 0.05 ml of 1% homogenate (w/v) according to the manual of SOD assay kit (Jiancheng Bioengineering Ltd, Nanjing, China). The absorbance of samples at 550 nm was detected with a spectrophotometer (722, Shanghai Jinghua Technology Instrument Co., Ltd). The protein content was measured by the method of coomassie brilliant blue.

Hippocampal and cortical MDA content were detected according to the manual of the maleic dialdehyde assay kit (Jiancheng Bioengineering Ltd.). After Morris water maze test, brains were removed (*n* = 6). The hippocampi and cortex were homogenized with normal saline. MDA content was detected using 0.2 ml of 10% homogenate (w/v). The absorbance at 532 nm was detected with the spectrophotometer. The protein content was measured by the method of coomassie brilliant blue.

### ELISA analysis

Quantification of PGE2 levels was carried out using cortex and hippocampi in an ELISA kits (TaKaRa Japan) according to the manufacturer's specifications.

### Statistical analysis

The results are expressed as the means ± standard deviation (SD) and were analyzed using SPSS 17.0 (SPSS Inc. Chicago, US). Within-group variances were compared using Dunnett's *t*-test.
